# Filling gaps in notification data: a model-based approach applied to travel related campylobacteriosis cases in New Zealand

**DOI:** 10.1186/s12879-016-1784-8

**Published:** 2016-09-06

**Authors:** E. Amene, B. Horn, R. Pirie, R. Lake, D. Döpfer

**Affiliations:** 1 Department of Medical Sciences, School of Veterinary Medicine, University of Wisconsin-Madison, Madison, USA; 2 Institute of Environmental Science and Research, Christchurch, New Zealand

**Keywords:** Campylobacteriosis, Bayesian specification, Multiple imputation, Missing value

## Abstract

**Background:**

Data containing notified cases of disease are often compromised by incomplete or partial information related to individual cases. In an effort to enhance the value of information from enteric disease notifications in New Zealand, this study explored the use of Bayesian and Multiple Imputation (MI) models to fill risk factor data gaps. As a test case, overseas travel as a risk factor for infection with campylobacteriosis has been examined.

**Methods:**

Two methods, namely Bayesian Specification (BAS) and Multiple Imputation (MI), were compared regarding predictive performance for various levels of artificially induced missingness of overseas travel status in campylobacteriosis notification data. Predictive performance of the models was assessed through the Brier Score, the Area Under the ROC Curve and the Percent Bias of regression coefficients. Finally, the best model was selected and applied to predict missing overseas travel status of campylobacteriosis notifications.

**Results:**

While no difference was observed in the predictive performance of the BAS and MI methods at a lower rate of missingness (<10 %), but the BAS approach performed better than MI at a higher rate of missingness (50 %, 65 %, 80 %). The estimated proportion (95 % Credibility Intervals) of travel related cases was greatest in highly urban District Health Boards (DHBs) in Counties Manukau, Auckland and Waitemata, at 0.37 (0.12, 0.57), 0.33 (0.13, 0.55) and 0.28 (0.10, 0.49), whereas the lowest proportion was estimated for more rural West Coast, Northland and Tairawhiti DHBs at 0.02 (0.01, 0.05), 0.03 (0.01, 0.08) and 0.04 (0.01, 0.06), respectively. The national rate of travel related campylobacteriosis cases was estimated at 0.16 (0.02, 0.48).

**Conclusion:**

The use of BAS offers a flexible approach to data augmentation particularly when the missing rate is very high and when the Missing At Random (MAR) assumption holds. High rates of travel associated cases in urban regions of New Zealand predicted by this approach are plausible given the high rate of travel in these regions, including destinations with higher risk of infection. The added advantage of using a Bayesian approach is that the model’s prediction can be improved whenever new information becomes available.

**Electronic supplementary material:**

The online version of this article (doi:10.1186/s12879-016-1784-8) contains supplementary material, which is available to authorized users.

## Background

Information originating from investigation of notified cases of an infectious disease has the potential to inform about the epidemiology and risk factors associated with the disease. Aggregating demographic and risk factor information from surveillance systems can help to set policy, monitor trends, and develop risk management options. However, the value of this information is often compromised by incomplete or partial information related to individual cases.

In New Zealand, cases of notifiable diseases are reported by general practitioners, laboratories and public health workers and the information is stored in the *EpiSurv* database. *EpiSurv* is operated by the Institute of Environmental Science and Research (ESR) on behalf of the Ministry of Health. A series of case report forms (https://surv.esr.cri.nz/episurv/index.php) are used to collect information about cases, disease diagnosis and clinical course, risk factors for the disease and case management.

Campylobacteriosis has been a notifiable disease in New Zealand since 1980. Data from notified cases are reported annually in surveillance summaries and have been analyzed for trends and to assess the effect of specific interventions [1, 2]. These analyses are primarily based on demographic information, since for a variety of reasons the risk factor information is not supplied for all cases. However, the value of complete information on cases has been demonstrated by a sentinel site study in the Manawatu region of New Zealand, which has made a special effort to complete risk factor reporting, alongside microbial subtyping [3].

In an effort to enhance the value of information from campylobacteriosis notifications in New Zealand, we have explored the use of models to fill risk factor data gaps. As a test case, we examined overseas travel as a risk factor for campylobacteriosis. Identifying the proportion of cases of campylobacteriosis where infection was acquired overseas is important to properly understand and measure domestic risk factors and the success of any risk management interventions [4]. International travel as a risk factor is important, as the rate of overseas travel by New Zealanders is high (e. g. 46 trips per 100 per year as compared to the international average of 14 per 100 in 2008) [5, 6]. However, whether (or not) cases had travelled overseas as a potential risk factor is reported for less than half of the notified cases of campylobacteriosis, and the reporting of this factor varies considerably across the 20 District Health Boards (DHBs) in New Zealand. One approach to adjusting for this lack of data, as currently used in annual surveillance reports, is to apply the proportion travel related from the campylobacteriosis cases for which the information is available to those cases lacking travel information. This approach estimates that approximately 7 % of campylobacteriosis notifications nationally over the period 2000 to 2010 were acquired overseas. However this information may be biased and does not fully reflect regional variation. As an alternative, we applied Multiple Imputation (MI) [7] and Bayesian Specification (BAS) [8] models, seeking to adjust rates of travel associated illness and fill data gaps using covariates derived from demographic characteristics and travel rates in the general New Zealand population.

## Methods

### Empirical data

#### Campylobacteriosis notifications

Campylobacteriosis notification records were obtained from the *EpiSurv* database [9]. All case notifications were completely anonymized to conceal the identity of individuals. The database registers a number of demographic and risk factor characteristics of the cases in addition to clinical features. Regional information is available per DHB in the campylobacteriosis notification data.

There were 121,764 notifications of campylobacteriosis in New Zealand reported between 2000 and 2010. Of these, most were culture confirmed (‘Confirmed’) or epidemiologically linked to confirmed cases or outbreak sources (‘Probable’) (Table 1). As there are no definitive results for the cases with a case status of ‘Under investigation’ and ‘Unknown’, we excluded them from the analysis resulting 119,375 cases for the primary dataset (sum of the first two columns in Table 1). Among 119,375 cases, 44,285 (37.1 %) had complete information for the travel section of the *EpiSurv* questionnaire, and 3107 (7 %) of cases with information for this section had completed short term international travel. Since 0.6 % of *Age* and 1.6 % of *Sex* observations were missing in the primary dataset, the associated records were excluded making the total number of cases available for analysis to become 116,721. The dependent variable used for our regression model was overseas travel status of the notified cases.

**Table 1:** Total number of campylobacteriosis notification in New Zealand residents categorized by information on overseas travel (2000–2010)

Travel status	Campylobacteriosis status				
	Confirmed	Probable	Under investigation	Unknown	Total
No	41617	60	52	416	42145
Unknown	74481	110	222	1653	76466
Yes	3100	7	7	39	3153
Total	119198	177	281	2108	121764

#### Explanatory variables

A number of explanatory variables was derived from the *EpiSurv* and *Statistics New Zealand* databases to construct a regression models for predicting missing travel status of notified campylobacteriosis cases. A complete list of predictor variables extracted from the notification and travelers’ database is shown in Table 2 and a detailed description is given in Additional file 1. While *Deprivation index*, *Urban* (population under urban influence) and *Travel Rate* are variables at a District Health Board (=DHB) level; *Age*, *Sex*, *Season* and *Intervention* (whether the case was recorded before or after 2006) are case specific variables.

**Table 2:** Description of variables in the New Zealand campylobacteriosis notification and short term international travelers’ datasets (2000–2010)

Variables	Details
Deprivation index	Categorical, 1–10 scale (1 = least deprived, 10 = most deprived)
Urban	Numeric, Proportion of DHB population under urban influence
DHB	Categorical, Residence District Health Board
Travel rate	Numeric, Residence DHB’s rate of short term international travel
Report date	Year of campylobacteriosis notification, 2000-2010
Age	Four categories; <5, 5–19, 20–65 and 65+ Years
Sex	Two categories; Male and Female
Season	Four categories; Spring (Sep-Nov), Summer (Dec-Feb), Autumn (Mar-May) & Winter (Jun-Aug)
Overseas travel	Three categories; Yes, No, Unknown (62 % of the cases did not have travel information.)
Intervention	A binary indicator variable to identify before and after the 2006 poultry intervention period.

*Notes*: *Deprivation index*, *Urban*, *DHB* and *Travel Rate* are DHB level variables, whereas *Report Date, Age, Season, Overseas Travel* and *Intervention* are measured at an individual case level

### Statistical methods

#### Logistic regression

Since our response variable (overseas travel status) is a binary variable (1 = yes, 0 = No), a logistic regression model was applied to the data. The generalized form of the logistic regression model is shown in eq. (1).

1 $$ \log \left(\frac{p\left(Y=1\right)}{1-p\left(Y=1\right)}\right)={\beta}_0+{\displaystyle {\sum}_{j=1}^k{\beta}_j{x}_j} $$

Where *p(Y = 1)* is the probability that a case made short term overseas travel and β’s are the regression coefficients, *k* = number of covariates (*x*’s). See Table 2 and Additional file 1 for the detail description of the covariates.

##### Missing At Random Assumption (MAR)

There are three types of missing data mechanisms, namely *Missing Completely At Random* (MCAR), *Missing At Random* (MAR) and *Missing Not At Random* (MNAR) [10]. MCAR occurs when the missingness is completely at random and results obtained from only completely observed cases can be used for inference, whereas MNAR indicates a systematic missingness in the data and requires explicit model for the missing data mechanism. The MAR scenario on the other hand also requires a model but can use measured covariates. MAR assumes that the probability of missingness only depends on the covariates in the data. In the MCAR and MAR situations, the missing data mechanism is frequently referred to as *ignorable,* i.e., we do not need a separate model for the missing data mechanism. In our case, the covariates obtained from *Statistics New Zealand* and the *Episurv* were used to construct the model. Most missing data methods including MI require this assumption to be fulfilled for a valid inference. While the MAR assumption, as such, is not statistically testable, it can be supported by demonstrating association of predictors with the missingness. We investigated this by fitting a logistic regression with dependent variable missingness of overseas travel (1 = missing, 0 = otherwise) on covariates. A statistically significant association indicates that the missingness can be explained by the covariates (i.e., the MAR assumption can hold.) A detailed description of types of missing data can be found in the literature [10, 11].

#### Multiple imputation

Multiple Imputation is a principled way of handling incomplete data where missing observations are replaced by draws from the predictive distribution of the missing data given the observed data [12, 13]. According to Rubin (1996), MI is a three-step process. First, sets of plausible values for missing observations are created. Each of these sets of values ‘fill-in’ the missing values (assuming MAR) and create multiple ‘complete’ datasets, so called ‘multiply’ datasets. Simulation studies have shown that as few as 3 ‘multiply’ datasets are adequate for a dataset with 20 % missing values [14]. Other studies have shown that 5–10 ‘multiply’ datasets are usually optimum depending on the proportion missing [7]. Second, each of these ‘multiply’ datasets can be analyzed using standard complete data methods. Finally, the results are pooled using Rubin’s rule, which allows the uncertainty regarding the imputation to be taken into account [15]. The *R* package *MICE* (Multiple Imputation using Chained Equations) was used for performing MI [7]. In this study, we have used 20 multiply datasets. We used the pooled regression coefficients to construct a logistic regression equation for predicting the probability of overseas travel.

All potential predictors available in our dataset were incorporated into the imputation model. Including all covariates predictive of overseas travel will help the MAR assumption to be increasingly plausible, in addition to producing unbiased results [16, 17]. This is because subjects with missing data based on (other) known characteristics, i.e. MAR- are by definition a random subset from the sample given these known characteristics (Table 1).

#### Bayesian Specification (BAS)

The Bayesian method allows to jointly use information coming from the observed data and from prior information on unknown parameters to derive inferences about missing data and parameters using Markov Chain-Monte Carlo (MCMC) algorithm [18]. While MI was derived from within a Bayesian framework (sampling from the posterior distribution of missing values, conditional on observed values), Bayesian approaches have been applied more generally [19]. Bayesian modelling provides a flexible method for incorporating different assumptions about the missing data mechanism and accommodating different patterns of missing data in the model [20]. For example, we can specify a separate model for the missing data mechanism if the information for estimating the missingness obtained (i.e., in the case of so called ‘informative missing response’) [21]. In our case, however, the data contain no information regarding the mechanism by which missing data were introduced and therefore we assume the missing data mechanism to be ignorable. In this case, the BAS treats missing data as additional unknown parameters and automatically generates values from its posterior predictive distribution for filling the missing data.

We used the *JAGS 3.4.0.* program (Just Another Gibbs Sampler) for Bayesian analysis, which is called into the R environment through *rjags* package [22]. The use of a Bayesian method requires that the priors of unknown parameters to be specified properly [23]. This is a way of incorporating uncertainty about the parameters into the model. For our analysis, all regression coefficients and the intercept were assigned uninformative priors (a normal distribution with mean 0 and standard deviation of 100, i.e. each with an inverse variance of 10^−4^) (Additional file 2) [8]. For computational reasons, Bayesian models in *JAGS* require the variance to be specified in terms of the precision (inverse of the variance). The models were run for 30,000 iterations with the first 3000 iterations discarded as burn-ins. All models were initialized with two chains. For realistic starting values, we set the initial values for each chain obtained from the fitted regression coefficients (see Additional file 2). As Bayesian inference relies on MCMC algorithm to draw samples from the posterior distribution, convergence of the algorithm has to be assessed, i.e., whether the Markov chains have reached a stable equilibrium distribution. Convergence indicates that the samples from the MCMC process are, in fact, drawn from the actual joint posterior distribution of the parameters. This was done through visually evaluating density plots, autocorrelation and the Brooks-Gelman-Rubin (BGR) statistic of the parameters in the models. The BGR statistic is a convergence diagnostic that compares the within and between chain variances where a value around 1 indicates convergence [23].

### Data analysis

#### Model development

First, we fit a multiple logistic regression model (*Eq.**2*) to the dataset containing *Complete Cases* (CC) (*n* = 44,285) using Frequentist and Bayesian frameworks. The CC analysis refers to analysis restricted to campylobacteriosis notifications with fully reported travel status (i.e., disregarding missing values). This subset of the original dataset included 38 % (44,285) of all notifications reported between 2000 and 2010. The remaining 62 % (72,436) lack travel information. The reason for performing this restricted analysis was to select the best prediction model based on cases with complete data.

2 $$ \begin{array}{l} \log \left(\frac{p\left(Y=1\right)}{1-p\left(Y=1\right)}\right)={\upbeta}_0+{\upbeta}_1*\mathrm{URBAN}+{\upbeta}_2*\mathrm{DEPRIVATION}+{\upbeta}_3*\mathrm{TRAVEL}+{\upbeta}_4*\mathrm{AGE}+\hfill \\ {} + {\upbeta}_5*\mathrm{SEASON} + {\upbeta}_6*\mathrm{SEX} + {\upbeta}_7*\mathrm{INTERVENTION}\hfill \end{array} $$

Next, we investigated the performance of MI and BAS for different rates of artificially introduced missing data to the CC (10 %, 50 %, 65 % and 80 % missingness on overseas travel status was introduced). In order to achieve the required percentage of missing values, we stratified the data into a cross tabulation based on two strata of the variable SEX and four artificial strata of the variable URBAN (i.e., $\le$ 0.6 = 1, 0.6-0.8 = 2, 0.8-0.9 = 3, >0.9 = 4). The SEX and URBAN variables were chosen for convenience. Then, we deleted the stratum (or strata) from the cross tabulation where the counts sum up to the desired proportion of missingness. Deleting specific strata from the dataset will ensure that the resulting missing data are MAR. We generated one sample per each category of artificially introduced missing data. Then, for every category, we fit a separate logistic regression model (Eq. 2) and summarized the outputs in Fig. 4. Finally, based on the models’ performance parameters on missing data prediction, we selected the best model and applied it for predicting overseas travel status in the original dataset.

#### Model evaluation and performance

We evaluated the performance of our models by comparing *Percent Bias (PB)* and *Brier Score (BS)* of regression coefficients and predictions, respectively. The *PB* indicates the percent deviation of the regression coefficients of models fitted to the missing data as compared to those estimated by the fully observed dataset (i.e. Complete Cases) (Eq. 3). Note that, the description of bias used here is slightly different to the usual definition (the expectation of difference between parameter estimates) [21].

3 $$ PB = \left({\beta}_m-{\beta}_f\right)/\left.{\beta}_f\right)*100 $$

where β_*f*_ is the regression coefficient estimated from the models fitted to the complete cases, and β_*m*_ is the regression coefficient estimated from the other models (i.e. using data including missing values). The BS, on the other hand, is an overall measure of predictive performance, i.e. a combination of discrimination and calibration [24] (Eq. 4). The *BS*, or average prediction error is defined as follows:

4 $$ \begin{array}{l}BS=\frac{1}{N}{\displaystyle \sum_{i=1}^N{\left({f}_i-{O}_i\right)}^2}\hfill \\ {}\kern2em i=1,\dots, N\hfill \end{array} $$

where *f*_*i*_ are predicted probabilities by the model, *O*_*i*_ is the observed outcome (0 or 1), and *N* is the total number of observations. A *BS* value close to 0 indicates the model performs well, whereas larger scores indicate poorly fitting models [25].

Additionally, we evaluated our models using the area under the receiver operating characteristic *(ROC)* curve. The *ROC* is often used to summarize and compare the discriminatory accuracy of a diagnostic test or modality, and to evaluate the predictive power of statistical models for binary outcomes [26]. We used the ROC curve analysis to evaluate how accurate our logistic regression models were in predicting overseas travel. Accordingly, we selected the *BAS* approach as a method of choice to apply to the original dataset.

#### Prediction of overseas travel

A Bayesian logistic regression model was fitted to the original dataset (*n* = 116,721) to predict missing overseas travel status of notified campylobacteriosis cases. The priors for all parameters in the model were specified as uninformative (see Additional file 2). We ran the sampler for 30,000 iterations and used 2 chains and 3000 iteration burn-ins. Finally, we investigated model fit by examining density plots, autocorrelation and trace plots of a subset of parameters in the model for a visual graphical assessment. After a convergence was achieved (i.e., after each chain mixed well and appeared stationary indicating that the target distribution was reached), we extracted the predicted summary measures of probability of overseas travel for individual cases (mean and standard deviation) from the posterior distribution. Since our main interest was to produce average predictions per reporting region (DHB), we summarized those individual predictions into a pooled mean $\left(\overline{\mu}\left({x}_i\right)\right)$ and SD per reporting region (*SD*_*j*_). To compute these values, we stratified the predicted probabilities by DHB, and then we calculated the mean (expected value) and the pooled SD per DHB, respectively, as shown in Eqs. 5 and 6 below.

5 $$ E\left({X}_j\right)=\overline{\mu}=\frac{1}{n}{\displaystyle {\sum}_{i=1}^n{x}_i} $$

where *DHB*_*j*_ (*j* = 1,2,…,20) consisting of *n* elements *x*_*1*_,..,*x*_*n*_ denoting individual predictions.

6 $$ S{D}_j=\sqrt{\frac{1}{n}{\displaystyle \sum_{i=1}^n\left({x}_i-\overline{\mu}\right)}+\left(\frac{1}{n}{\displaystyle \sum_{i=1}^n{\sigma}_i^2}\right)} $$

where *SD*_*j*_ denotes the pooled SD for *DHB*_*j*_*, x*_*i*_ = individual predictions, ${\overline{\mu}}_j$ =the mean prediction for *DHB*_*j*_, *σ*_*i*_^2^ = the variance of individual predictions and *n* = number of observations per DHB.

## Results

**Fig. 1:**
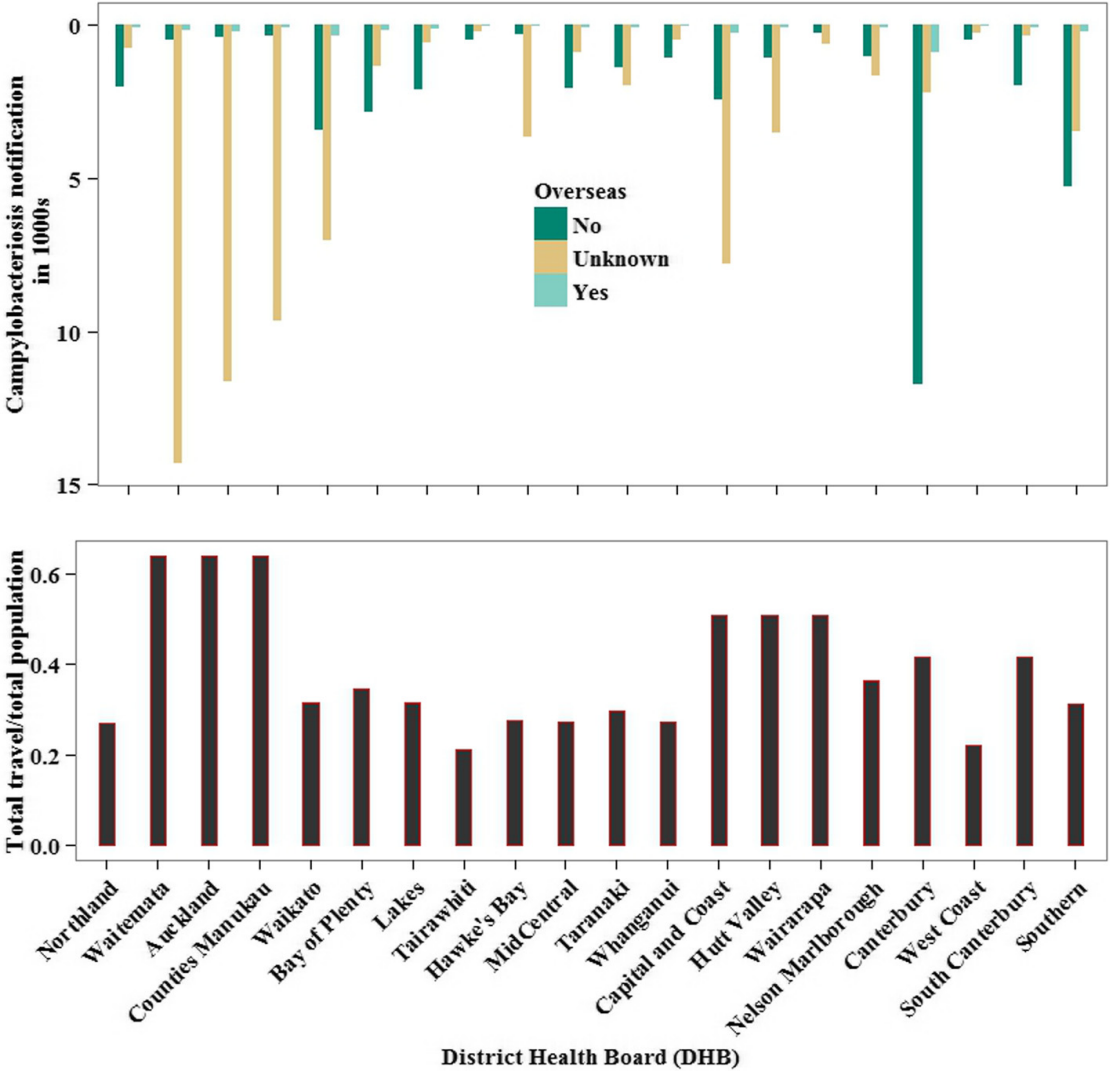
Distribution of campylobacteriosis notification categorized by the status of overseas travel (upper panel) and the annual proportion of short term international travels (lower panel), in DHBs of New Zealand (2000 – 2010). *Notes*: Upper panel: campylobacteriosis notification in 1000s is the sum of all cases notified between 2000 and 2010 in a given District Health Board; lower panel: Total travels/total population: the average number of outbound travels per year divided by the average population size per year between 2000 and 2010 for a given District Health Board

Figure 1 displays the total number of notified Campylobacter cases between 2000 and 2010 which are categorized by the status of overseas travel reporting and annual rate of overseas travels per person in each DHB. Most of the cases reported from Auckland, Waitemata, and Counties Manukau DHBs lack travel information. However, the majority of reported cases and more than 55 % of all travel between 2000 and 2010 originated from residents in these DHBs [27]. As shown in Fig. 1, more than 60 % of all cases come from six DHBs, namely Waitemata (12.8 %), Canterbury (12.7 %), Auckland (10.6 %), Waikato (9.3 %), Capital and Coast (8.9 %), and Counties Manukau (8.7 %) (Fig. 1).

**Fig. 2:**
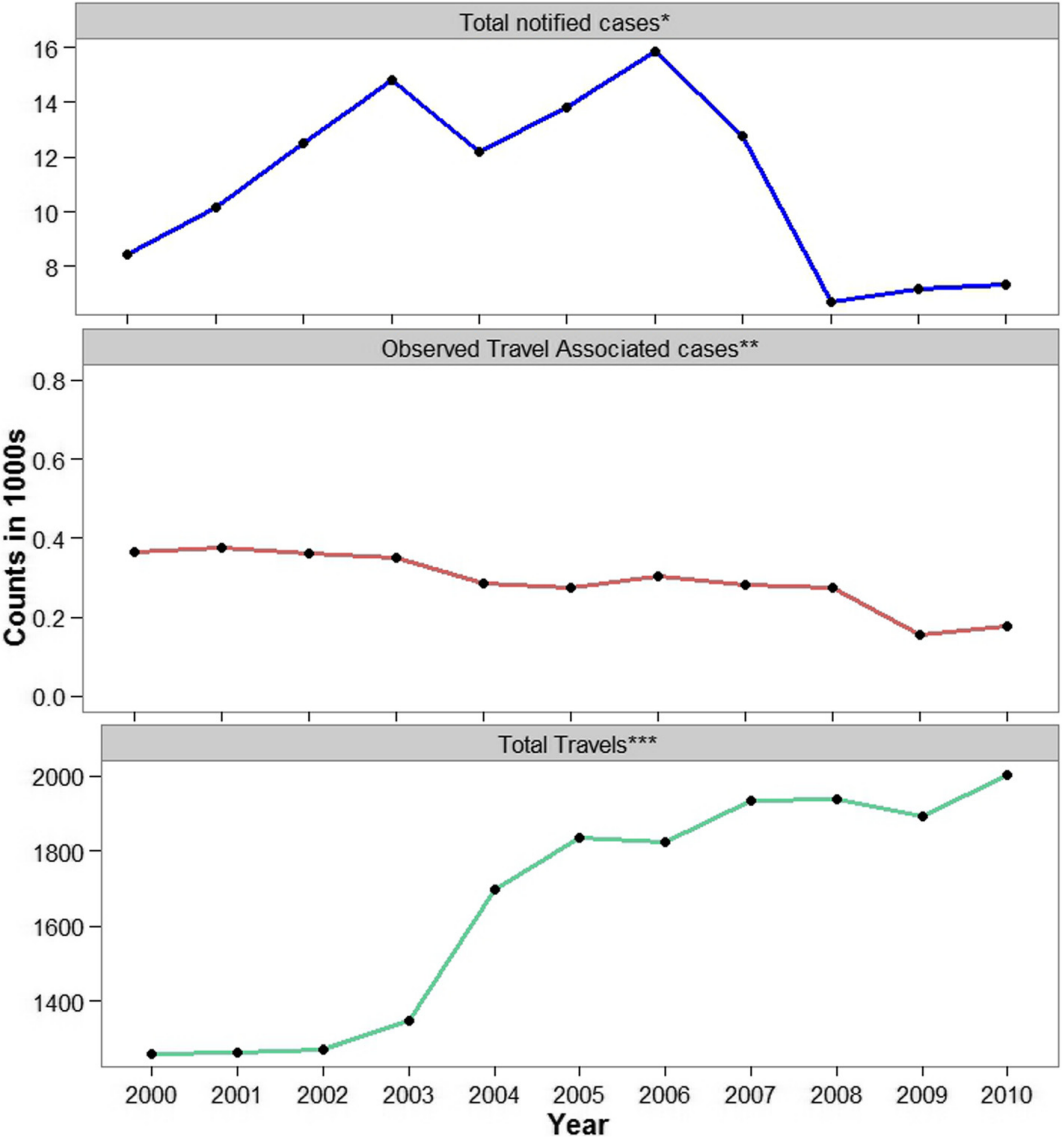
Annual short term international travel and campylobacteriosis notification of New Zealand residents (2000–2010). *Total notified cases: total number of campylobacteriosis cases notified between 2000 and 2010. **Observed travel associated cases: campylobacteriosis cases that had confirmed overseas travel during the incubation period of the disease. ***Total travels: total number of short term international travels between 2000 and 2010. Short term international travel is defined as international departures of New Zealand residents for an intended period of less than 12 months (*Statistics New Zealand* [www.stats.govt.nz])

**Fig. 3:**
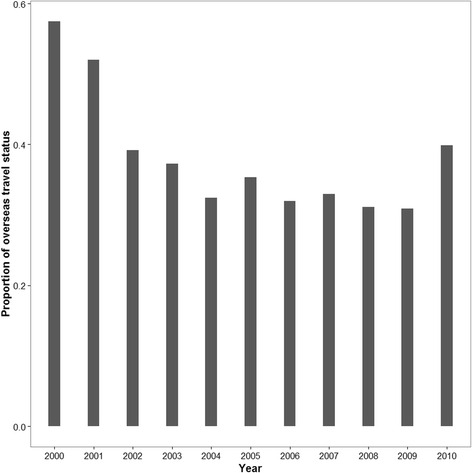
The proportion of campylobacteriosis notifications in New Zealand with known status of overseas travel information (2000–2010)

The number of short term international trips by New Zealanders consistently increased between 2000 and 2010 (bottom panel in Fig. 2). As evident from Fig. 2, total campylobacteriosis notification in New Zealand had been increasing until 2006 except a slight decrease in 2003–2004. After 2006, the total number of notifications declined significantly. The total number of reported travel associated cases and the overall trend of availability of information on travel status for the notified campylobacteriosis cases have declined over time except a slight increase in 2010 (see the middle panel of Fig. 2 and Fig. 3).

Table 3 shows the result of statistically examining the association between predictors and whether or not overseas travel was missing using the original dataset (*n* = 116,721). Majority of the predictors are strongly associated with missingness in overseas travel implying that missingness in the data can be explained by the fully observed variables in the model which supports the MAR assumption (Table 3).

**Table 3:** Summary of logistic regression analysis for variables predicting missing indicator (1 = missing overseas travel information, 0 = otherwise) to test the validity of Missing At Random assumption (n = 116721)

Coefficients	Estimate	Std. Error	Pr(>|z|)
(Intercept)	−8.757	0.089	<0.001
Urban^a^	2.992	0.103	<0.001
DepIndex^b^	0.525	0.006	<0.001
Travel Rate^c^	0.081	0.001	<0.001
Age (5–19)	0.154	0.027	<0.001
Age (20–59)	0.033	0.023	0.145
Age (60+)	−0.142	0.027	<0.001
Summer	0.014	0.018	0.443
Autumn	−0.002	0.021	0.94
Winter	0.035	0.021	0.085
Male	0.153	0.014	<0.001
Intervention^d^	0.345	0.016	<0.001

*Keys*: ^a^Proportion of DHB population under urban influence; ^b^Deprivation index (scale 0–10, 0 being least deprived and 10 being most deprived DHB; ^c^Short term international travel per 100 residents of a DHB; ^d^A binary indicator variable to identify pre and post 2006 intervention. Age (<5), Spring, and Female sex are reference categories

**Fig. 4:**
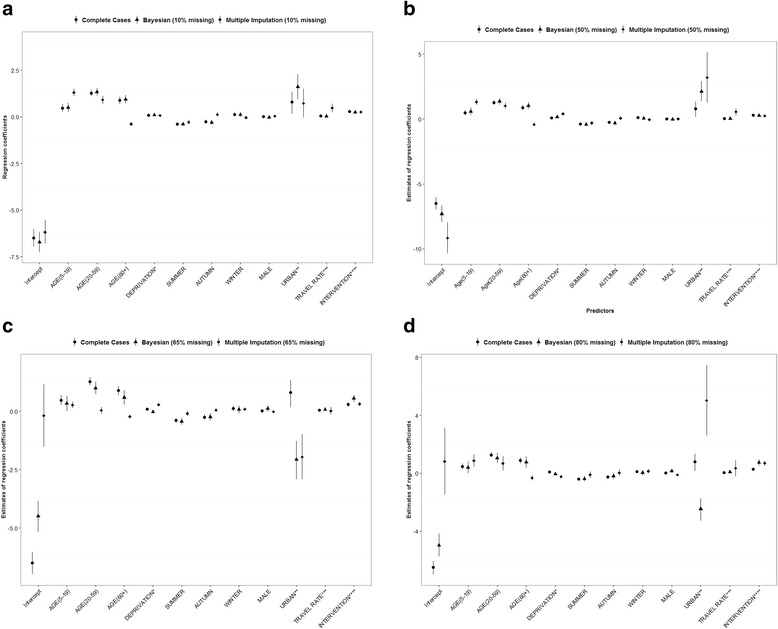
Comparison of Bayesian and Multiple Imputation models regarding the mean and 95 % Credibility (Confidence) Intervals of regression coefficients for 10 % (Fig. 4a), 50 % (Fig. 4b), 65 % (Fig. 4c) and 80 % (Fig. 4d) missing data category as compared to the complete data on overseas travel status of campylobacteriosis cases (*n* = 44,285). *Notes*: (1) * Deprivation index (scale 1–10, 1 = least deprived and 10 = most deprived District Health Board; **proportion of DHB population under urban influence;*** Short term international travel per 100 residents of a DHB; ****a binary indicator variable to identify cases that were reported before or after 2006 poultry intervention period. (2) Complete cases: regression coefficients estimated from campylobacteriosis notification data with complete information on overseas travel. (3) The error bars indicate the 95 % confidence intervals (in Multiple Imputation models) and 95 % Credibility Intervals (in Bayesian models) of the regression coefficients

The outcomes of applying MI and BAS models to the datasets with artificially induced missingness is given in Table 4 and Fig. 4. Comparison of BS and AUC to select the best predictive model shows that the BAS model is more robust than MI as the rate of missingness increases (Table 4). At 10 % MAR, there was no difference between MI and BAS. However at 50 %, 65 % and 80 % MAR cases, the BAS approach resulted in relatively higher AUC and smaller BS than MI (Table 4). Furthermore, results of the model outputs (i.e., mean and 95 % uncertainty bounds of the regression coefficients) for all categories of missing data as well as the outputs from the complete cases are presented in Fig. 4a, b, c and d. There was no remarkable difference in the regression coefficient estimates across the four categories of artificial missing data. However, most of the regression estimates and 95 % CIs of the BAS model are closer to the values estimated using complete cases as compared to the estimates from the MI model (Fig. 4). This evidence suggests that, the BAS model performs relatively better for a dataset with a high rate of missing values. In addition, no significant difference between the regression coefficient estimates was observed from the Bayesian model fit to original dataset (*n* = 116721) and to the CC dataset (*n* = 44,285) (see Table 5).

**Table 4:** Comparison of Brier Score and Area Under the Curve (AUC) between Bayesian and Multiple Imputation models for the prediction of overseas travel status of campylobacteriosis cases

Accuracy measure	Complete data^a^		Missing data^b^							
	Frequentist	Bayesian	Multiple Imputation				Bayesian			
			10 %	50 %	65 %	80 %	10 %	50 %	65 %	80 %
Brier Score	0.062	0.062	0.067	0.24	0.18	0.19	0.062	0.063	0.062	0.063
AUC^c^	0.67	0.67	0.64	0.49	0.42	0.49	0.67	0.67	0.65	0.64

^a^ *n* = 44,285

^b^Four categories of artificially introduced missing data (10 %, 50 %, 65 % and 80 % missing overseas travel status)

^c^Area Under the Receiver Operating Characteristic Curve

**Table 5:** Summary of logistic regression coefficients for the original dataset containing missing observations (*n* = 116,721) and the Complete Cases dataset (*n* = 44,285) using Bayesian models

Coefficients	Original dataset^a^		Complete Cases^b^			
	Mean	95 % CI^2^		Mean	95 % CI	
Intercept	−6.503	−6.965	−6.041	−6.522	−6.978	−6.070
Urban^c^	0.804	0.231	1.377	0.834	0.297	1.414
DepIndex^d^	0.091	0.063	0.119	0.091	0.063	0.120
Travel Rate^e^	0.045	0.040	0.051	0.045	0.039	0.050
Age (5–19)	0.473	0.262	0.683	0.476	0.270	0.680
Age (20–59)	1.273	1.095	1.452	1.278	1.105	1.449
Age (60+)	0.885	0.688	1.082	0.889	0.697	1.080
Summer	−0.393	−0.491	−0.294	−0.393	−0.491	−0.297
Autumn	−0.254	−0.364	−0.143	−0.255	−0.367	−0.145
Winter	0.128	0.027	0.230	0.128	0.026	0.229
Male	0.015	−0.060	0.090	0.015	−0.059	0.089
Intervention^f^	0.288	0.200	0.377	0.287	0.199	0.377

^a^All campylobacteriosis notifications available for analysis (n = 116,271); ^b^campylobacteriosis notifications containing information on overseas travel status (n = 44,285). ^c^ Proportion of DHB population under urban influence; ^d^Deprivation index (scale 0–10, 0 = least deprived and 10 = most deprived DHB); ^e^Short term international travel per 100 residents of a DHB; ^f^A binary indicator variable to identify pre and post 2006 intervention. Age (<5), Spring, and Female sex are reference categories

**Fig. 5:**
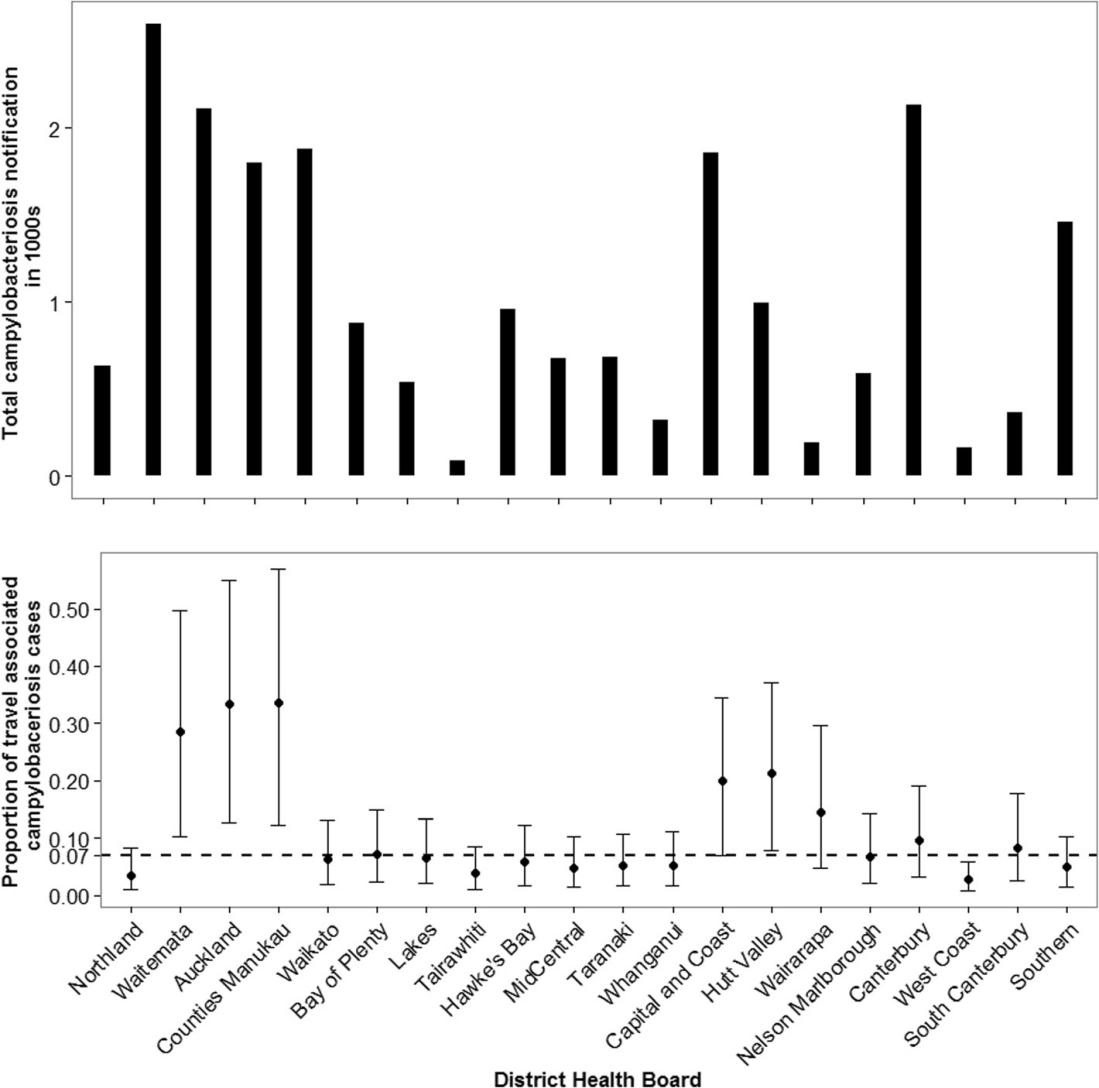
The total number of campylobacteriosis notification (upper panel) and the proportion of travel related cases predicted by the Bayesian model (lower panel) for each DHB of New Zealand (2008–2010). Notes: (1) Bottom panel: proportion of travel related cases predicted by the Bayesian model. The error bars are 95 % Credibility Intervals of the proportion of overseas travel. (2) The dashed horizontal line is the proportion of travel related campylobacteriosis cases for which travel history is available nationally (7 %)

The BAS model was applied to the original dataset to estimate the proportion of cases due to overseas travel in each DHB during the period 2008–2010. During this period the number of campylobacteriosis notifications and travel rates were relatively stable. Figure 5 shows the total number of notified campylobacteriosis cases (upper panel) and the estimated proportion of travel related cases as predicted by our model (lower panel). The horizontal dashed line in the bottom panel is drawn to indicate the percent of reported travel associated cases (7 %) among all cases that have provided travel information.

In many of the DHBs with a high rate of campylobacteriosis notification (see upper panel of Fig. 5) and high rate of travel (see bottom panel of Fig. 1), such as Auckland, Counties Manukau and Waitemata, our model predicted a high proportion of campylobacteriosis cases to be associated with overseas travel. For example, the proportion of travel associated cases was higher in Counties Manukau, Auckland and Waitemata DHBs, at 0.34 (0.12, 0.57), 0.33 (0.13, 0.55) and 0.28 (0.10, 0.49), whereas the lowest proportions were estimated for West Coast, Northland and Tairawhiti at 0.02 (0.01, 0.06), 0.03 (0.01, 0.08) and 0.04 (0.01, 0.08) respectively. Except for Auckland, Counties Manukau, West Coast and Waitemata DHBs, the 95 % CI of the predicted proportion of travel associated cases included the observed national proportion of travel related cases (horizontal dashed line in bottom panel of Fig. 5). Accordingly, the national estimate and 95 % CI of the proportion of travel related cases based on our model is 0.16 (0.02, 0.48).

## Discussion

Data gaps in notification data have been a continuous public health challenge for identifying the source of infection and preventing infectious diseases, including campylobacteriosis. The increase of overseas travel by New Zealanders and the established risk of overseas travel for *Campylobacter* infection emphasize the need to study travel associated illnesses.

A total of 18.3 million short term international trips by New Zealand residents were recorded between 2000 and 2010. Most travel was to the Pacific region, East Asia and North America, while the least travel was recorded for the regions of West and Central Africa and Antarctica. This is in agreement with previous reports that New Zealanders travel to more than 150 countries, of which countries in the Pacific region and North America are the most popular destinations [6]. In the meantime, international travel has been increasing in New Zealand since 2004 (see Fig. 2). In contrast, a substantial reduction of incidence of notified campylobacteriosis cases occurred after 2006 (Fig. 2). The significant changes in notifications post 2006 were believed to be the result of interventions targeting poultry [2]. Despite this overall decline in notifications of campylobacteriosis in New Zealand, the change attributable to cases associated with overseas travel is not well understood. Although the outbound travel rate of New Zealand residents has been increasing, we noticed a decline in notified travel associated cases throughout the study period except a slight increase in 2010 (middle panel of Fig. 2). This could be due to the corresponding decrease in reporting of travel information for the cases throughout the study period (Fig. 3) that may have confounded conclusions on the origin of the disease.

In addition, there is a consistently low reporting rate of detailed travel information in urban areas of New Zealand such as in Auckland and Wellington regions. A case control study in the New Zealand regions with high notifications rates, including Auckland region, suggests that recent overseas travel was a significant risk factor for the occurrence of campylobacteriosis in this region [28].

The majority (62 %) of campylobacteriosis case reports in New Zealand lack travel history during the incubation period prior to disease. The level of completeness of travel history for notified cases has been a challenging task as is reported by some other studies [29–31]. It is therefore necessary to estimate travel associated cases based on imperfect data.

Among the total number of notifications with known travel history, only 3107 (7 % of notifications with known travel status) had travelled overseas during the incubation of the disease. As New Zealanders are prolific travelers, this proportion of cases may underestimate the true contribution of travel as a risk factor for campylobacteriosis in New Zealand. For this reason, model-based methods such as MI and BAS can be useful to fill the data gaps, using covariates that predict overseas travel. The use of BAS and MI methods provides a methodology to calculate uncertainty bounds around the estimates of travel associated cases. The degree of uncertainty of the predicted proportion of travel associated cases can be attributed to variation in the risk of travel associated illnesses among individuals that have different covariate values. Such variation in the risk of campylobacteriosis with respect to age, sex and season is in agreement with previous reports in literature [5, 32].

The BAS model resulted in an estimate of the national proportion of notifications due to overseas travel of 16 %, a higher value compared to 7 % estimate using only known values. Similar or higher rates of travel related campylobacteriosis have been reported in other developed countries such as in Canada (21.6 %) [33], England (17 %) [29], USA (18 %) [34], Denmark (18 %) [35] and Switzerland (46.1 %) [36].

Our model predicted a high proportion of travel associated cases in major urban areas of New Zealand, such as in Auckland, Counties Manukau and Waitemata DHBs. This could be due to high rates of travel of their residents to the Pacific Islands and South East Asia regions, which is partially driven by the comparatively high proportion of Asian ethnicity (23.8 %) and Pacific Peoples (14,6 %) in the Auckland region [6, 27]. It has been previously established that individuals traveling to these world regions are at a higher risk of travel associated illnesses, including campylobacteriosis [37]. On the other hand, the DHBs with a smaller proportion of model-predicted travel related cases (e.g., Northland, West Coast and Tairawhiti) are those with a lower outbound travel rate.

If the MAR assumption holds, which is usually difficult to achieve, our Bayesian model provides a plausible way for predicting missing overseas travel of campylobacteriosis cases [20]. It is also important to note that any other missing data analysis approaches require assumptions that are just as difficult to justify [11]. At the same time, the BAS procedure should not be viewed as the ‘gold standard’ for filling data gaps for every situation, although it offers a flexible approach for data augmentation. Priors can be enhanced if data regarding association of risk factor–outcome become available. In addition, the Bayesian model specification can be modified if the missing data mechanism is non-ignorable and the missingness model can be verifiable [38].

Better notification reporting, particularly for areas with high outbound travel and high notification of cases such as in highly urban areas can improve our understanding of the epidemiology of travel associated campylobacteriosis in New Zealand. However, reporting completeness is limited by the resources available in each DHB. Use of alternative data collection approaches such as web based applications, cross tabulation of Customs data with *EpiSurv* data, and creating awareness in the population regarding the importance of the information for the public health databases may improve reporting completeness. Although the emphasis in this report is on predicting travel information of *Campylobacter* cases in New Zealand, the method can be implemented for other diseases of public health significance which have similar data gaps.

## Conclusion

The common challenge of data gaps regarding risk factors for campylobacteriosis suggests the use of model-based approaches for estimating missing values. Filling data gaps is particularly important for regions with a high rate of incomplete data. The Bayesian modelling approach offers a flexible alternative for data augmentation particularly when the missing rate is very high.

## Additional files

Additional file 1: The description of explanatory variables used for predicting overseas travel status of campylobacteriosis notification in New Zealand [2, 27, 39]. (DOCX 16.3 kb) Additional file 2: JAGS code for the Bayesian Hierarchical model [22, 40]. (DOCX 17.5 kb) Additional file 3: Number of campylobacteriosis notifications and overseas travels in New Zealand District Health Boards (DHBs) (2000-2010). (XLSX 11 kb)

## Abbreviations

AUC: Area under the curve
BAS: Bayesian specification, BS, Brier score
CC: Complete case
CI: Credibility interval
DI: Deprivation index
ESR: Environmental sciences research
MAR: Missing at random
MCAR: Missing completely at random
MCMC: Markov Chain Monte Carlo
MI: Multiple imputation
MICE: Multiple imputation using chained equations
MNAR: Missing not at random
PB: Percent bias
SD: Standard deviation
