# Supplementary material for: Filling gaps in notification data: a model-based approach applied to travel related campylobacteriosis cases in New Zealand
Source: BMC Infect Dis. 2016 Sep 6;16(1):475. doi: 10.1186/s12879-016-1784-8 (PMC5011939; doi:10.1186/s12879-016-1784-8)
Supplement: Additional file 1: — The description of explanatory variables used for predicting overseas travel status of campylobacteriosis notification in New Zealand [2, 27, 39]. (DOCX 16.3 kb) [file 12879_2016_1784_MOESM1_ESM.docx]

**Appendix 1: The description of explanatory variables used for predicting overseas travel status of campylobacteriosis notification in New Zealand**

***Overseas Travel***

A custom data extract for short term international travel of New Zealand residents between 2000 and 2010 was obtained from Statistics New Zealand [1]. The data included the travel patterns of New Zealand residents to international destinations including; countries visited, month of arrival back in New Zealand and demographic structures of travelers. Short term travel is defined as international departures of New Zealand residents for an intended period of less than 12 months. The annual number of overseas trips for a region was calculated at the DHB level. The total number of trips during the study period (2000-2010) was divided by the average DHB population estimates during the same period as extracted by Statistics New Zealand [1] to provide a population rate (TRAVEL RATE) which was used in the modelling.

***Urban or rural lifestyle***

Statistics New Zealand [1] used seven categories to define urban and rural New Zealand in 2006: *main urban*, *satellite urban*, *independent urban*, *rural area with high urban influence*, *rural area with moderate urban influence*, *rural area with low urban influence* and *highly remote/rural area*. The 2006 census data has been analyzed by Statistics New Zealand to provide population estimates in each of these urban and rural categories. The proportion of a DHB’s population living in one of the three urban areas, i.e. *main urban*, *satellite urban* and *independent urban* (URBAN) is used as a DHB level variable in the modelling. The *main urban* areas are those having a minimum population of 20,000 and the *satellite urban* areas identifies towns and settlements with strong links to main urban areas through employment. The *independent urban* areas on the other hand are those whose population is without a significant dependence on main urban centers through employment location [1].

***Deprivation Index***

*New Zealand Deprivation Index (DI)* is a measure of socioeconomic deprivation which combines certain variables from the 2006 census reflecting eight dimensions of deprivation. These include income, home ownership, need of support, employment, qualifications, the amount of living space, access to communication and access to transport [2]. *DI* is expressed in an ordinal scale ranging from 1 to 10 (1=least deprived, 10=most deprived) [2]. It is a small-area measure of deprivation, which implies that, *DI* score was provided for a subgroup of population within a *DHB.* In order to get one composite measure for *DI* to be incorporated as a covariate for modeling at the *DHB* level (*DEPRIVATION*), we weighted the *DI* score by the proportion of the population corresponding to the *DI* score. The result was a weighted median of the *DI* for the entire DHB population as shown below.

Let *D_j_* = {*d_1_*, *d_2_*,…, *d_n_*} be a set of ordered deprivation scores (n=10) for *DHB_j_*, and *W_j_* = {*w_1_*, *w_2_*,…, *w_n_*} be the corresponding weights such that $\sum_{i=1}^{n} w=1$ (i.e., the proportion of the DHB population corresponding to the deprivation score) for *DHB_j_*,

then, the weighted median is the element *d_k_* satisfying

$\sum_{d_{i} < d_{k}} w_{i}<1/2 and \sum_{d_{i}> d_{k}} w_{i}\leq1/2$

***Poultry Intervention***

In 2007 interventions were put into place by the New Zealand Poultry Industry to reduce *Campylobacter* in retail poultry alongside the introduction of a regulatory *Campylobacter* Performance Target. This resulted in a drop in campylobacteriosis notifications from 15,728 in 2006 to 6,594 in 2008 [3]. As a domestic intervention, the total decrease in campylobacteriosis cases would not have affected the number of cases resulting from overseas travel. A binary variable (INTERVENTION), which identifies cases reported either before or after 2007, was added to the model to allow the sudden drop in notifications to be incorporated into the model.

***Season***

As a Southern Hemisphere country, New Zealand’s seasons are the inverse of North America and Europe. Below is description of the four seasons:

*Spring*: September, October and November

*Summer*: December, January and February

*Autumn*/Fall: March, April and May

*Winter*: June, July and August

For incorporating as a covariate in the regression modeling, season variable (SEASON) was derived from the notification and travel databases based on months of reporting.

***Age***

Both the *EpiSurv* and *Statistics New Zealand* records age ranges of notified campylobacteriosis cases and short term international travelers, respectively. The *EpiSurv* records age of cases in 5 and 10 year intervals (< 4, 5-9, 10-19,30-39, 40-49, 50-59, 60-69 and 70+) whereas Statistics New Zealand records age of travelers mostly in a 5 year interval (<4, 5-9, 10-14, 15-19, 20-29, 25-29, 30-64 and 65+). In order to use *age* as a covariate in the models, we re-categorized it into four groups (<5, 5-19, 20-64 and 65+) in both datasets (*AGE*). This will facilitate the merging of the two databases for subsequent modeling.

**Appendix references**

1. Statistics New Zealand. Home - Statistics New Zealand. [Accessed 2014 Apr 10]. Available from: http://www.stats.govt.nz/

2. Salmond C, Crampton P, Atkinson J: NZDep2006: New Zealand Index of Deprivation, 2007.

3. Sears A, Baker MG, Wilson N, Marshall J, Muellner P, Campbell DM, Lake RJ, French NP: Marked campylobacteriosis decline after interventions aimed at poultry, New Zealand. Emerg Infect Dis 2011, 17:1007–1015.
