# Supplementary material for: Filling gaps in notification data: a model-based approach applied to travel related campylobacteriosis cases in New Zealand
Source: BMC Infect Dis. 2016 Sep 6;16(1):475. doi: 10.1186/s12879-016-1784-8 (PMC5011939; doi:10.1186/s12879-016-1784-8)
Supplement: Additional file 2: — JAGS code for the Bayesian Hierarchical model [22, 40]. (DOCX 17.5 kb) [file 12879_2016_1784_MOESM2_ESM.docx]

## Additional file 2: JAGS code for the Bayesian Hierarchical model

The JAGS code used for estimating travel associated campylobacteriosis cases in New Zealand is presented below. All the parameters in the model were given uninformative prior distributions (i.e., were assigned dnorm(0, 0.0001) priors, which implies a normal distribution with mean = 0 and precision =10^-4^). Two chains of dispersed initial values were ran each with 30,000 iterations and a burn-in of 3000. Model convergence was assessed by observing the mixing of the two chains and visually checking the density plots and autocorrelation.

modelCampyTravel = "

model {

for(i in 1:N) {

OvseasCat [i] ~ dbern(p[i]) **# Likelihood**: observed travel status of individual cases is Bernoulli distributed

p[i] <- 1/(1+exp(-(β_0_ + β_1_*URBAN[i]+ β_2_*DEPRIVATION [i]+ β_3_*TRAVEL [i]+ β_4_ [AGE[i]]+ β_5_[SEASON[i]]+ β_6_[SEX[i]]+ β_7_[INTERVENTION[i]])))

} **# Predicted values**

β_0_~dnorm(0, 0.0001)  **# Uninformative priors on regression coefficients**

β_1_~dnorm(0, 0.0001)

β_2_~dnorm(0, 0.0001)

β_3_~dnorm(0, 0.0001)

β_4_ [1]<-0 **# Set first categories of factors to 0 (reference categories)**

β_5_ [1]<-0

β_6_ [1]<-0

β_7_ [1]<-0

for (b in 2:4) **# Uninformative exchangeable priors for categorical predictors**

{ β_4_[b]~dnorm(0, 0.0001) }

for (a in 2:4)

{ β_5_[a]~dnorm(0, 0.0001) }

β_6_[2]~dnorm(0, 0.0001)

β_7_[2]~dnorm(0, 0.0001)

}

"

writeLines(modelCampyTravel,con="model.txt")

modelCheck( "model.txt" ) **# Check the model**

**# Define data**

Data <- list(

N=length(data$OvseasCat), OVERSEASCAT = data$OvseasCat, URBAN = data$urban,

DEPRIVATION= data$DEPRIVATION, TRAVEL= data$TRAVEL, AGE=data$AGE,

SEASON=data$SEASON, SEX=data$SEX, INT=data$INT

)

**# Parameters to monitor**

parameters = c("β_0_","β_1_","β_2_","β_3_", "β_4_","β_5_","β_6_", "β_7_", "p")

**# Initial values**

Inits<-list(

list(β_0_=-6.52+.5, β_1_=0.83]+.1, β_2_=0.09+.1, β_3_=0.05+.1, β_4_=c(NA, rep((0.47+.1),3)), β_5_=c(NA, rep((-0.25+.1),3)), β_6_=c(NA,0.02+.1), β_7_=c(NA, 0.29+.1)),

list(β_0_=-6.52-.5, β_1_=0.83]-.1, β_2_=0.09-.1, β_3_=0.05-.1, β_4_=c(NA, rep((0.47-.1),3)), β_5_=c(NA, rep((-0.25-.1),3)), β_6_=c(NA,0.02-.1), β_7_=c(NA, 0.29-.1)),

)

# **set.seed (123)**

**# MCMC settings**

ni<- 30000 **# Number of draws from posterior**

nb<- 3000 **# Number of draws to discard as “burn-in”**

nc <- 2 **# Number of chains to run**

**# Gibbs sampling**

jags.fit <- jags(data= Data, inits=Inits,

parameters.to.save=parameters,

model.file="model.txt",

n.chains=nc,

n.iter=ni,

n.burnin=nb)

# ***Notes:*** *OvseasCat*: Overseas travel status (YES, NO, UNKNOWN), *ProU*: Proportion of the population under urban influence, *DepIn*: Deprivation Index, *TrvlRate*: DHB’s international travel rate, *age*: age of travelers, *season*: season of travel, *sex*: sex of travelers.

*JAGS* (Just Another Gibbs Sampler): is a standalone program for simulating from a Bayesian Hierarchical models that takes a model string written in an R-like syntax and that compiles and generates a Monte Carlo Markov Chain (MCMC) samples from this model using the Gibbs sampling algorithm [1]. The main advantage of *JAGS* over the classical *BUGS* (Bayesian Inference Using Gibbs Sampling e.g., *WinBUGS*) is its platform independence that it can operate in all main operating systems, while *BUGS* is broadly Windows specific. *JAGS* is called and controlled within the R environment through *rjags* package [2]. It parameterizes distributions using precision instead of standard deviation (σ), where the precision *τ = 1/* σ^2^. Therefore in the above JAGS code, the standard deviation of the prior distribution becomes:

0.0001 =1/100^2^

**References**

1. Plummer M, others: JAGS: A program for analysis of Bayesian graphical models using Gibbs sampling. In *Proceedings of the 3rd international workshop on distributed statistical computing*. *Volume 124*. Technische Universit at Wien; 2003:125.

2. Plummer M, Stukalov A, Denwood M, Plummer MM: Package **“**rjags**.”** *update* 2015, 16:1.
